# Chancre of the Gum

**Published:** 1886-11

**Authors:** 


### SELECTIONS.

### Chancre of the Gum.

Dr. D. N. Kinsman thus writes in the *Cleveland Medical Gazette*:

The peculiar mode of infection in the following seems to justify its publication. While there is, of course, an element of doubt, such as must exist in all such cases, I feel sure its origin is such as I shall detail.

B. consulted me June 24 conserning a swelling of the cervical and sub-maxillary glands on the right side. They were large, hard, painless, and without the adhesions to the surrounding tissues usual in the ordinary cases of adenitis of their duration.

In seeking for a cause for their development I found an ulcer surrounding the right middle incisor, in the upper jaw. This ulcer had been under treatment by a dentist for several days, and by him was supposed to be due to an accumulation of "tartar,"

which had extended beneath the gum and caused the ulceration. It had been treated with iodine locally.

I told him the glandular swellings were due to the ulcer, and when it healed the swelling would disappear. He returned to the dentist for treatment for a few days. I saw him again July 1. There was no change in the aspect of the case, except the glandular swelling was increased, and the glands of the other side of the neck and those in the occipital region were also involved.

I found he had fever and headache, for which I gave quinine. July 3 he was worse and sent for me to see him at home. At this visit the case became clear, for the papular syphilide upon his face, chest, and arms left no possible doubt. He subsequently had nocturnal pains in the head and shins, and pharyngitis and patches upon the tongue. Under specific treatment he did well, and in five weeks the apparent manifestations had disappeared. Three years ago this man was treated for supposed syphilis. This diagnosis seems to have rested upon the occurrence of a sore upon the penis and a consecutive suppurating bubo. He had none of the secondary accidents of syphilis.

For some time the origin of this chancre was a mystery. Finally, I learned that B. was having sexual relations with a young woman who was living at home, and that they had continued for more than a year. In the early part of May, or last of April, this young woman, who till then had been free from all sores, had a fissure on her lip, which was cauterized with a pencil of nitrate of silver. This fissure took on the appearance of a broad-based sore, so the young man says, and for some time refused to heal. Finally, the glands in her neck enlarged. She had an eruption, and, upon consulting a physician, the case was diagnosticated syphilis, and accordingly treated, when her symptoms subsided.

B. supposed that he had been poisoned when kissing her while her lips were sore, and I am led to believe, from all I can learn, this woman was contaminated by the pencil of silver, which may have been soiled by contact with a chancre on some one else. Such modes of infection are common enough. This case, as well as others, teaches us we cannot be too careful to protect others from contamination by soiled instruments which have been used about syphilitics.

